# Melanoma and Nevi Subtype Histopathological Characterization with Optical Coherence Tomography

**DOI:** 10.3390/life13030625

**Published:** 2023-02-23

**Authors:** Cristina L. Saratxaga, Aintzane Asumendi, Jesús Gardeazabal, Rosa M. Izu, Ana Sanchez, Goikoana Cancho-Galan, Celia Morales, Sergio Lage, Maria D. Boyano, Olga M. Conde, Estibaliz Garrote

**Affiliations:** 1 TECNALIA, Basque Research and Technology Alliance (BRTA), Parque Tecnológico de Bizkaia, C/Geldo. Edificio 700, 48160 Derio, Spain; 2 Photonics Engineering Group, University of Cantabria, 39005 Santander, Spain; 3 Department of Cell Biology and Histology, Faculty of Medicine and Nursing, University of the Basque Country/Euskal Herriko Unibertsitatea (UPV/EHU), 48940 Leioa, Spain; 4 Biocruces Bizkaia Health Research Institute, 48903 Barakaldo, Spain; 5 Dermatology Department, Cruces University Hospital, 48903 Barakaldo, Spain; 6 Dermatology Department, Basurto University Hospital, 48013 Bilbao, Spain; 7 Pathology Department, Basurto University Hospital, 48013 Bilbao, Spain; 8 Valdecilla Biomedical Research Institute (IDIVAL), 39011 Santander, Spain; 9 CIBER-BBN, Biomedical Research Networking Center—Bioengineering, Biomaterials, and Nanomedicine, Avda. Monforte de Lemos 3–5, Pabellón 11, Planta 0, 28029 Madrid, Spain

**Keywords:** skin cancer, melanoma, OCT, HE, optical biopsy, histopathology, optical properties, textural properties, CADx

## Abstract

Background: Melanoma incidence has continued to rise in the latest decades, and the forecast is not optimistic. Non-invasive diagnostic imaging techniques such as optical coherence tomography (OCT) are largely studied; however, there is still no agreement on its use for the diagnosis of melanoma. For dermatologists, the differentiation of non-invasive (junctional nevus, compound nevus, intradermal nevus, and melanoma in-situ) versus invasive (superficial spreading melanoma and nodular melanoma) lesions is the key issue in their daily routine. Methods: This work performs a comparative analysis of OCT images using haematoxylin–eosin (HE) and anatomopathological features identified by a pathologist. Then, optical and textural properties are extracted from OCT images with the aim to identify subtle features that could potentially maximize the usefulness of the imaging technique in the identification of the lesion’s potential invasiveness. Results: Preliminary features reveal differences discriminating melanoma in-situ from superficial spreading melanoma and also between melanoma and nevus subtypes that pose a promising baseline for further research. Conclusions: Answering the final goal of diagnosing non-invasive versus invasive lesions with OCT does not seem feasible in the short term, but the obtained results demonstrate a step forward to achieve this.

## 1. Introduction

Melanoma is the deadliest form of skin cancer and the 17th most common cancer worldwide [1]. Incidence has increased in the latest decades due to sun exposure and radiation, immunosuppressant medicines, infections with papilloma viruses, genetics, etc., and it is expected to increase worldwide by 50% in 2040 (510,000 new cases) with according to the 2020 statistics (~325,000 new melanoma cases) [2]. Consequently, optical biopsy of dermatological lesions is long being pursued by dermatologists in the latest years as a non-invasive complementary tool to clinical examination and dermatoscopy. The greatest limitation that dermatologists claim is the discrimination of melanoma from benign nevi and, more concretely, the differentiation of non-invasive from invasive lesions. Considering that from 9% to 58% of melanoma (mostly invasive) originate from a nevus [3,4], this differentiation becomes crucial in the early stage of diagnosis in clinical practice. 

To respond to this need, the scientific community has studied different imaging techniques, wherein optical coherent tomography (OCT) stands out because of its capacity to provide an overview of tissue morphological architecture and lateral lesion delimitation. Various works studying the application of OCT in the diagnosis of melanoma have been published in the latest years, but as recent review studies confirm [5,6,7,8], there is still a lack of agreement on proposals and differential criteria on images’ visual interpretation, and further studies with more number of samples are still necessary. The main weaknesses of OCT that prevent its use in clinical practice are the limited view of deep lesions, the misleading diagnosis of benign and malignant lesions (amelanotic melanoma diagnosed as basal cell carcinoma), and the lack of clinicians with training on image interpretation. With the aim to overcome this, different evolutions of conventional OCT are under research, such as: line-confocal OCT (LC-OCT) [9], dynamic OCT (D-OCT) [10,11], and high-definition OCT (HD-OCT) [12]. Some latest solutions advocate for the combination of OCT with other techniques. For example, a combination with ultrasound (US) provides an overall visualization of more extensive lesions (sacrificing image resolution) and complementary molecular information can be provided by Raman spectroscopy [13]; however, all of these multimodal approaches are still in the initial steps of development. Unfortunately, none of these solutions have overcome the mentioned problems during diagnosis. 

Morphological and histopathological features and other characteristics previously reported in the literature can be further studied and exploited to improve and assist the automatic interpretation of OCT images. For example, it is known [14] that benign nevi present an increased number of melanocytes; however, although grouped in nests, they maintain their size. On the contrary, in the case of melanoma, there is not only an increase in the number of melanocytes, but they also become atypical and larger and are more frequently seen on the epidermis in a pagetoid spreading pattern. In [15], image-related observed features are presented. Correlation to histopathological features associated with melanoma has also been targeted [16] (and even some newly proposed), such as the identification of pagetoid spread, atypical melanocytes, architectural disorder at the dermoepidermal junction (DEJ), invasion of tumour cells into the dermis, etc. In other approaches, some new features have also been identified and associated with melanoma and nevi subtypes or phases [17], such as the presence of shadows (in-situ melanoma), shadow and loss of bright collagen (invasive melanoma), or hypo-reflective band (compound nevi). 

Additionally, OCT images can be studied from an optical point of view, so the information contained can be automatically highlighted with the support of artificial intelligence solutions and used to assist clinicians during diagnosis or excision. In this sense, the first attempts were reported in [18], where different in vivo optical properties were quantified and conclusions were made that the “relative attenuation factor of the first layer (Lraf1)”, the “skin entrance signal (SES)”, and “half value layer (z1/2)” were associated with a bigger success in the diagnostic accuracy of melanoma in comparison to only accounting morphological (histopathological) features. They reported differences in the attenuation factor of the epidermis when comparing malignant melanoma, dysplastic nevus, and benign nevus. The OPE (optical properties extraction) algorithm has been presented in [13] and continues to evolve in [19], where tissue-scattering coefficient, absorption coefficient, and anisotropy factor optical properties were calculated from OCT images and integrated, and then signatures associated to melanoma were identified in a trained machine learning approach. Additionally, they proposed an “optical radiomic melanoma detection (ORMD)” protocol that is aimed to be used by clinicians during diagnosis, providing hints about the tissue similarity with melanoma or a healthy status. Some others [20] have also suggested analysing the images from a signal point of view with fractal dimensions (FD) using 2D Fourier fractal analysis and differential box counting method for the classification of lesions. A decrease of 2D FD was hence found for benign melanocytic nevi with respect to melanomas, and hence, FD is used to extract the abnormality of biological tissue that happens in the presence of melanoma. 

Current status of OCT adoption in daily dermatological clinical routine demonstrates that there is still some necessary work to be done. Considering the diagnostic limitations of the technology, this work intents to propose an alternative path of application and analysis where the goal is to make the most of the information that is provided by OCT images (even with lower performance equipment). For this reason of attending the medical needs, the focus is being put in the discrimination of non-invasive versus invasive lesions assisted by automatic image processing techniques (and in the future, advanced machine learning techniques) in comparison with previously mentioned works that target the differentiation of all benign versus all malignant lesions relying on the clinician’s image interpretability capacity. Therefore, this work presents a detailed comparative analysis of features extracted from OCT images with respect to the gold-standard haematoxylin–eosin (HE) histopathology images examined by a pathologist followed by the comparison of a series of optical and textural features that are applied to the samples with the aim to investigate if some differentiation of lesions can be observed. In the future, this knowledge could be integrated into Computer Aided Diagnosis (CADx) tools to assist dermatologists in the optical diagnosis of lesions with OCT, diminishing the subjectivity in current image interpretation. As a starting point, with the support of pathologists and dermatologists, some specific cases have been selected for analysis and some hypotheses have been raised to be proven or discarded when analysing the results after feature extraction and histopathological comparison. 

Section 2 presents materials and methods, where the equipment is described, the clinical procedure for the collection of samples depicted, samples’ histopathological features described, selected case studies from the dataset presented, diagnosis hypothesis presented, and characterization and procedure for feature extraction detailed. Then, Section 3 presents a comparative analysis of the main results, whereas Section 4 discusses the results and remarks on the conclusions of this work. Additionally, Appendix A includes the haematoxylin–eosin (HE) histopathological images of the selected case studies and a table summarizing the main characteristics.

## 2. Materials and Methods

### 2.1. Equipment

**Figure 1:**
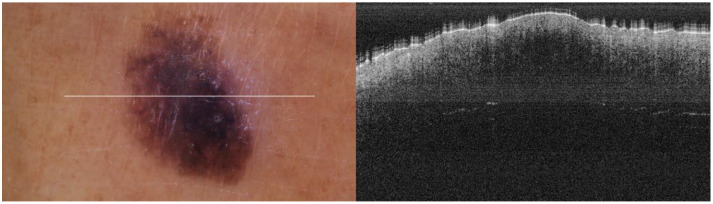
Example of dermatoscopy and OCT images from the equipment. White line in dermatoscopy image represents the scanning path (9 mm) of the OCT image (superficial spreading melanoma Case 2).

The portable NITID device developed by Medlumics (Madrid, Spain) [21] was used in the clinical settings due to its transportation ease and utilization in two different hospital premises. This equipment integrates miniaturized technology that allows acquiring clinical, dermatoscopy (14 mm field-of-view (FOV), 1.16 mpx image resolution, 20–40× magnification, 1920 × 1080 pixels image size), and OCT images (selectable FOV from 3 to 12 mm, 12 μm lateral resolution, 11 μm axial resolution, up to 1.9 mm in depth, 425–1700 × 512 pixels image size). Dermatoscopy images integrate a white line in the middle that indicate the approximate scanning path of the OCT image (B-scan) based on the selected FOV. The OCT works at 1300 nm and is based on a time–domain configuration, providing a scanning speed of up to 24 kHz. Figure 1 shows an example of both images for a melanoma lesion case.

### 2.2. Sample Acquisition Clinical Procedure

Patients were recruited at Cruces University Hospital and Basurto University Hospital (Spain) prior to obtaining informed consent. Data and images were collected with the approval of the Euskadi Clinical Research Ethics Committee (CEIC) and the hospitals’ ethical committees. Targeted lesions during the acquisition period were melanoma and nevus. Two technicians trained in OCT image acquisition were assigned to each hospital. Patients were examined with the equipment after consultation with the dermatologist. OCT performance time at each hospital was limited to one day per week to minimize the impact on their clinical routine due to the extra time required to perform image acquisition (~20 min). 

**Figure 2:**
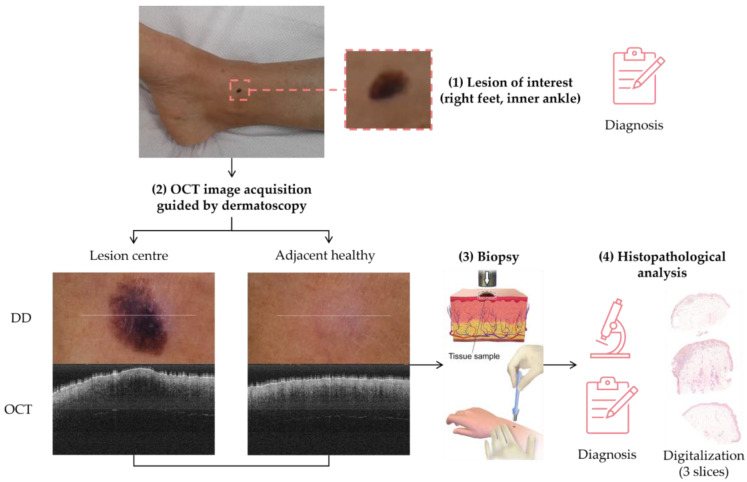
Clinical procedure followed in the hospitals for each database sample acquisition (superficial spreading melanoma Case 2).

The sample acquisition process that led to the creation of the dataset followed the steps illustrated in Figure 2. First, a suspicious lesion was identified by the dermatologist, and dermatological diagnosis was delivered plus additional clinical information was gathered. Second, the lesion was analysed with the OCT device, and the image acquisition was guided by the dermatoscopic image provided by the device. The most convenient FOV was selected (based on lesion size) with the aim of ensuring that the OCT images of the lesion should include healthy tissue on both sides of the lesion. As a result (as illustrated in Figure 2), an OCT image taken with the centre of the lesion (with adjacent healthy tissue on both sides) and its corresponding dermatoscopic image, jointly with OCT and dermatoscopic images of totally healthy tissue near the lesion were obtained. In the event of an extensive lesion, the lesion was acquired in parts, ensuring that the lesion’s OCT images include both the lesion and adjacent healthy tissue at least on one side. Third, a biopsy was performed and tissue sample was extracted. Then, fourthly, the sample was processed for histopathological analysis. Three different slices of the sample were extracted (after paraffin fixation) and stained with HE: one in the centre and two on the sides between the centre and the end of the sample. These slices were analysed by the pathologist, and the final diagnosis of the lesion was established (which may differ from the initial dermatologist’s diagnosis). Next, selected slices were scanned, making digital histopathological images available for comparison and study.

Summing up, for each lesion of interest, the following information was collected: clinical image, clinical data, dermatologist’s diagnosis, dermatoscopy images (lesion and nearby healthy tissue), OCT images (lesion in the centre and adjacent healthy tissue on the sides and nearby healthy tissue), histopathological diagnosis, and HE digital images.

### 2.3. Samples’ Histopathological Features

**Figure 3:**
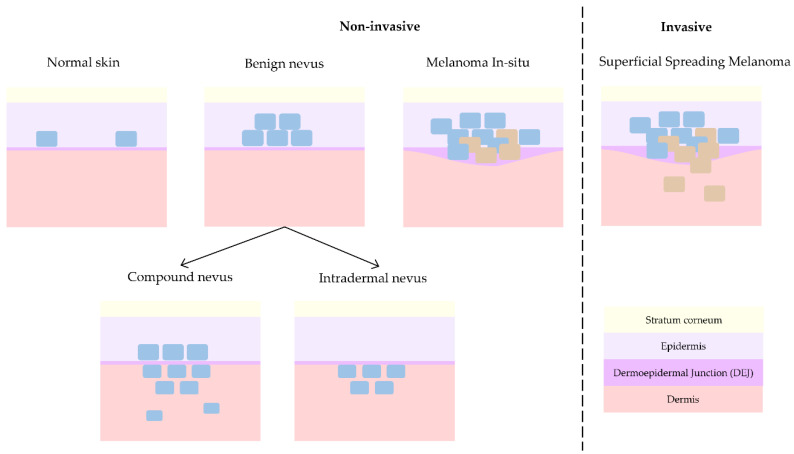
Progression of skin lesions: from normal skin to non-invasive benign nevus and melanoma in-situ and finally invasive melanoma.

Melanoma is the deadliest form of skin cancer and hence where main research interest lies. As mentioned before, melanoma can either evolve from a pre-existing nevus [3,4] or grow spontaneously. This is the reason why the differentiation with respect to nevus (differential diagnosis) is typically pursued. However, dermatologists collaborating in this study (J.G., R.M.I., A.S.) consider the discrimination of non-invasive lesions versus invasive lesions is the key issue in daily clinical practice [22]. Compound nevus (CN), junctional nevus (JN), intradermal nevus (IN), and melanoma in-situ (MIS) are considered non-invasive lesions, whereas superficial spreading melanoma (SSM) and nodular melanoma (NM) are considered invasive. When analysing histopathological features, in the case of MIS, as depicted in Figure 3, tumour cells grow horizontally (radially) and remain inside the epidermis, facilitating the treatment of local excision of the lesion. On the other side, in the case of invasive melanoma, tumour cells can grow either horizontally or vertically (axially), invading the dermis layer and hence increasing the chances of metastasis with the consequent impact on patient prognosis. 

JN is a form of nevus that grows in the DEJ, although it can be so subtle that the resolution of the OCT equipment can be insufficient for its detection, and hence, collected cases have been discarded from this work to avoid misleading the interpretation of the results. On the other hand, NM usually presents with clinical (external) features that makes its recognition easier during patient examination for dermatologists. Besides, its invasive growth is vertical and not horizontal, as in the case of non-invasive melanoma in-situ or invasive superficial spreading melanoma, which clinically are the most critical cases to differentiate from each other. Since only one case of NM was obtained at the hospitals during the study time, it has been discarded for further analysis in this work. Therefore, this work only considers CN, IN, MIS, and SSM cases for advanced analysis with the main goal of differentiating non-invasive from invasive lesions. Figure 3 synthetizes the main differences among them from a histopathological point of view, showing the progression from healthy skin (with some isolated melanocytes in the epidermis) to an invasive melanoma (with uncontrolled growth of melanocytes in the dermis and trespass of tumour cells into the dermis). 

Table 1 indicates the layers where histological alterations occur for each type of lesion. CN occurs when tumour cells are located both in the epidermis and in the dermis. They are symmetrical lesions that present with a combination of junctional and intradermal components, wherein the borders of the lesion have junctional nevus characteristics and the centre’s characteristics are representative of compound nevus. Considering this, lateral border delimitation in OCT images raises a big issue, and it will mostly fail considering that the technology does not provide cellular level resolution necessary to appreciate junctional nevus cells as mentioned before. Histologically, cells are grouped in nests that can be observed at different depths. Nevus cells, which are melanocytic cells that mature and become smaller in depth, are the main characteristic cells that distinguishes this type of nevus from melanoma (dermal component). However, single nevus cells are not expected to be observed in OCT images due to its resolution, but it can acquire the effect of the groups of nests and its changes in the architecture and texture of epidermis and dermis layers, besides the loss of the DEJ. However, these changes that are expected to be seen in OCT images will depend on the stage of development of the nevus, where initial and moderate cases’ features will be more difficult to identify. In the case of IN, the tumour cells are only located in the dermis, and the DEJ integrity is maintained; however, OCT images reveal changes in DEJ’s usual shape. In the dermis, the IN and CN become different because in CN, melanocytes appear both attached to the upper dermis (as in the case of IN) and also falling/maturing in depth.

As summarized in Table 1 and Figure 3, a melanoma is considered in-situ (MIS) when tumour cells remain and grow horizontally in the epidermis, what is also considered Stage 0. Histologically, it is characterized by the appearance of nests and pagetoid spreading, which should be appreciated in OCT images as a change in tissue texture and granularity with respect to the healthy epidermis. SSM, which is the most common type of melanoma, occurs when the tumour grows by spreading horizontally along the skin surface (epidermis) but invasion to dermis and inner layers also occurs. Histologically, it also presents with nets, pagetoid spreading, and isolated tumour cells, which should again be distinguishable in OCT images. In general, from a histopathological point of view, the main difference between nevus and melanoma is that nevi are typically symmetric and mature in depth. Additionally, melanoma cases present with atypia in the malignant tumour cells and inflammatory response (lymphocytes and macrophages) acting against them (and in some advanced cases even ‘eating’ the tumour lesion). As both types of lesions usually present with pigmentation (melanin both in the epidermis and dermis layers), it makes it difficult to identify relevant features during diagnosis with the dermatoscope; thus, most suspicious lesions are biopsied and sent for detailed histopathological analysis and diagnosis. Unfortunately, the amount of melanin is also expected to interfere with OCT imaging of lesions, as the pigmentation will absorb an important part of the light and hence less features/elements/alterations may be observed in the images. 

### 2.4. Case Studies

Image acquisition during consultation time was not always easy (due to patients’ movements due to nervousness or age), which led to the appearance of artifacts in the OCT images in addition to those that appear in the presence of hair, injury, or blood or those intrinsic to the technology itself [23]. In some scenarios, the large presence of artifact made it impossible to use any of the images of the sample, which ended up being discarded from the dataset. Besides, comparison with HE slides was a challenging endeavour. As explained in the previous section, both the OCT image and the HE slides were taken from the middle of the lesion to facilitate image comparison. However, identical comparison cannot be guaranteed, and indeed desirable co-registration of images was not possible. Given this, a small subset of comparable samples were selected for further study. The reason behind it was that the cutting direction of the paraffined biopsies could not be controlled, and in most cases, the direction did not match (was not parallel) with the corresponding OCT images. All the collected images were reviewed one by one, until some cases were selected for the “correlation” of the OCT image with the corresponding HE image in a stochastic manner. 

The dermatoscopic and OCT images from the eight case studies included in this work are provided in Table 2 (CN cases), Table 3 (IN cases), Table 4 (MIS cases), and Table 5 (SSM cases) for consultation. These tables also include information about the lesion localization in the patient’s body as additional information to be potentially considered during the analysis. The corresponding HE images can be reviewed in Appendix A. This appendix also includes a table, defined with the support of a pathologist, summarizing the main histopathological features and alterations found in the lesions relevant for the analysis. 

### 2.5. Target Diagnosis Hypothesis 

Dermatologists demand new technologies and tools that support them in differentiating non-invasive and invasive lesions. Hence, a series of questions were proposed and expected to be answered through the detailed analysis of OCT images with features extraction dedicated algorithms and comparison with HE images. Note: The answers to these questions cannot be generalised to any OCT device but limited to the device used in this study.

**Question** **1.** *Are non-invasive and invasive lesions distinguishable?*

**Question** **2.** *Are melanoma in-situ (MIS) and superficial spreading melanoma (SSM) distinguishable?*

**Question** **3.** *Are superficial spreading melanoma (SSM) and compound nevus (CN) distinguishable?*

**Question** **4.** *Are melanoma in-situ (MIS) and intradermal (IN) nevus distinguishable?*

Being able to answer positively some of the previous questions would be a big step towards the final goal. From an image analysis point of view, solving Question 3 and Question 4 are most interesting. Question 3 is important because both lesions present alterations in epidermis and dermis, but their histopathological features and architecture are different, which should be reflected in OCT images to some extent and image analysis algorithms should be able to discover somehow. Besides, solving this question would have a high impact on the clinical side. Question 4 is relevant because lesions are located in different skin layers and algorithms should be able to discover this fact. Although answering Question 1 and Question 2 would solve current medical needs, solving Question 3 and Question 4 would create a solid algorithm baseline for future improvements and development. Question 1 is the target, but at this point is not expected to be solved in the short-term, considering the high histopathological heterogeneity of lesions. Finding some clues to answer Question 2 would also pose automatic image analysis proposals in the good direction for answering Question 1. 

### 2.6. Lesions Characterization & Feature Analysis

Since the goal of this work was to lay the foundations for building a CADx tool for clinicians, optical and textural properties were studied to find variations that may assist their differentiation. Hence, the first step was to perform an automatic delimitation of lesion’s lateral margins, so that features inside the lesion could be studied and compared when needed with healthy tissue present in adjacent areas or near the lesion. Then, a Region-Of-Interest (ROI) was extracted to limit posterior analyses to the area where relevant features were found and to discard ‘empty’ areas that would only include noise to the analysis. Finally, both optical properties and textural features were extracted and compared for the different lesion and diagnostic subtypes as stated in Table 1.

#### 2.6.1. Automatic Lesion Delimitation

**Figure 4:**
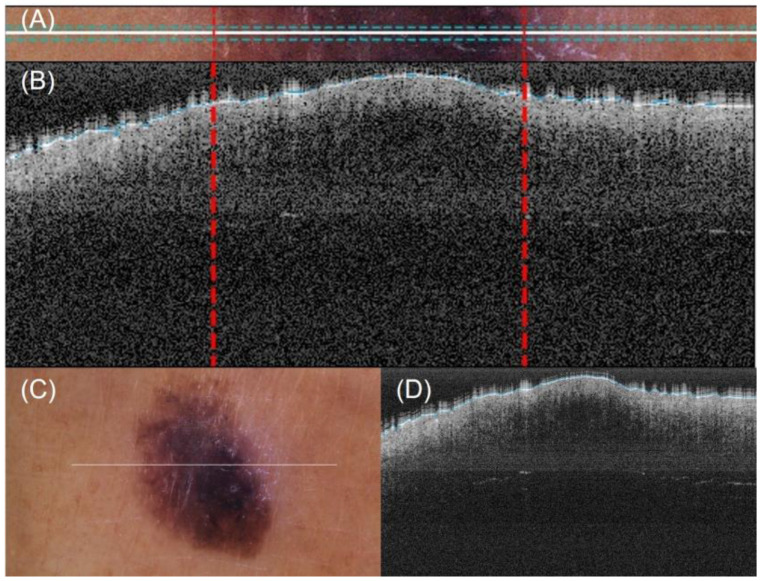
Automatic OCT lesion delimitation process: (**A**) zoomed image of lesion with lateral delimitation based on the colour of the dermatoscopic image; (**B**) delimitation transfer to OCT image with dotted red lines indicating delimitation; (**C**,**D**) original dermatoscopic and OCT images presented in Figure 1 (superficial spreading melanoma Case 2).

A method for automatic lateral delimitation of lesions in OCT images based on the dermatoscopic image proportionated by the equipment was previously described in [24]. This method uses the white line present in the dermatoscopic image (which approximately corresponds to the OCT scanning path) as the starting point. Then, an upper and a lower surrounding area of 10 px was added to obtain a sub-image (Figure 4A), where a strategy based on Otsu thresholds [25] was implemented to find the limits of the lesion. Finally, the last step performed the correct co-registration of the dermatoscopic sub-image and the OCT images to establish the lateral delimitation of the lesion, so that further analysis could be automatically performed within the area (Figure 4B). This lateral delimitation did not pursue to be a perfect delimitation of the lesion (based on external features, as internal can be different) but an approximation of the lesion localization to facilitate further automatic analysis of the OCT images as described later.

#### 2.6.2. ROI Extraction 

**Figure 5:**
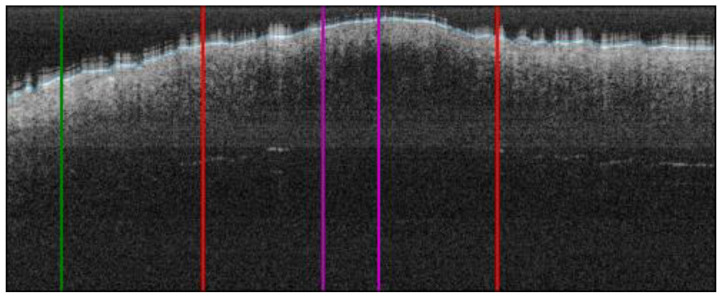
ROI extraction process: red bars indicate the automatic lesion delimitation as illustrated in Figure 4; purple bars delimitate the ROI at the centre of the lesion that is ROI considered for further properties extraction; green bar delimitates the healthy adjacent ROI starting on the left of the image (alternatively on the right) (superficial spreading melanoma Case 2).

Given the lateral delimitation of the lesion had been calculated in the previous step, the start and end of the lesion were established in the OCT image. Then, the centre of the lesion was calculated and 50 px was extracted along the left and right side, obtaining a lesion ROI of 100 px width (~0.7 mm). By default, we considered the centre of the lesion as the most representative area of the given diagnosis. This did not necessarily correspond to what happened histologically in all the possible cases, but it could be applied to the selected cases under the study to establish a generalized criteria for all the OCT images of the lesions under study. Additionally, another 100 px ROI of adjacent healthy tissue was also automatically extracted from the left or right side of the lesion (or both sides) depending on the number of pixels available. An example is illustrated in Figure 5. 

**Figure 6:**
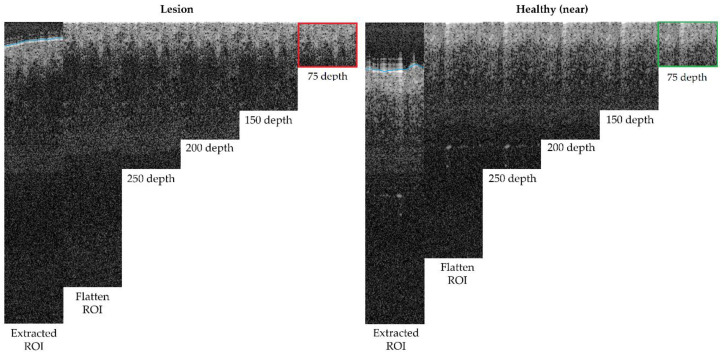
Depth analysis comparison for optimum size selection (superficial spreading melanoma Case 2).

The next step was to remove the air gap over the lesion. All the OCT images were roughly annotated to indicate the start of the tissue (blue line in OCT samples images, as in Figure 5), as artifacts were present in the image of a fully automated method and were not reliable. Then, this annotation was read for the area within the ROI and corrections were made when necessary. By default, the annotation delimitation was considered as the tissue starting point. Then, for each A-Scan (column), all the peaks found were analysed, and if a very high peak was found at the very beginning of the signal (corresponding to the brighter area where the laser light hits with the skin), the peak position was considered as the new starting value (index 0 value) of the A-scan. Hence, a flattened ROI was obtained, where the “air” and “shape” (overelevation) of the lesion was eliminated as it was considered superfluous information that could potentially alter or lead to erroneous results. The results can be observed in Figure 6, where the first column shows the initial ROI and second column (and beyond) the flattened ROI.

Finally, the last step was performed to limit the area in depth that is considered for the ROI, to be, afterwards, focused on the epidermis and dermis layer alterations and lesion architecture. After studying (Figure 6) different possible depths within the ROI area after flattening, it was observed that after 150 px (~0.4 mm), there was no valuable tissue information in the OCT image that might have contributed to the features to be extracted, but that most features were concentrated before 75 px (~0.2 mm). This happened for various reasons, mainly due to the penetration capacity of the OCT equipment into the tissue but also, for example, due to the pigmentation level of the lesion (as described in Table A1). 

#### 2.6.3. Optical Property Study

The estimation of the optical properties of tissues [26,27] is a valuable tool for the interpretation of OCT images that may help to improve the diagnosis of melanomas [14,18]. In OCT image analysis, when a single dispersion mode of light is considered, the intensity of light at distance z has been modelled following the Beer–Lambert equation [28,29]:

(1) $$I\left(z\right){=I}_{o}{\;e}^{-u_{t}z}\;$$

where I_o_ is the initial optical intensity and µ_t_ the total attenuation coefficient. The attenuation of the tissue is defined by the sum of absorption and scattering coefficients: µ_t =_ µ_s +_ µ_a._ Absorption refers to the fraction of laser light that the medium will absorb as it goes thought it. As regard to skin, the medium’s main absorbent components are melanin and haemoglobin [30]. As most of the melanoma and nevi lesions of the case studies were highly pigmented, the absorption was expected to be high. Scattering, on the other hand, refers to changes in the direction of the light inside the tissue that happens when the optical properties of small elements/components are different from those around. In the skin, scattering is associated with melanin (in dermis), collagen (in dermis), cell nuclei, cells walls, etc. [30]. 

For each A-scan in the extracted and flattened ROI sub-image, the attenuation coefficient µ_t_ was estimated through a Least Squares fit where 2 stands for the round trip of the double path followed by the signal:

(2) $$I\left(z\right){=A\;2\;e}^{-u_{t}z}+C$$

However, first, pixel dimension in the depth Z axis must be corrected. The theoretical penetration capacity of the equipment in air changes when interacting with a medium, skin tissue in this case, so its refraction index must be taken into consideration. To perform these calculations, the single refractive index value of human skin of n = 1.4 at 1300 nm was considered [31,32]. Then, since the equipment had a maximum penetration depth of 1.9 mm, corresponding to 512 pixels in the image, the real resolution of pixels in depth was calculated as follows:

$$\Delta z=\frac{\frac{1.9\;\times \;10^{3}}{n}}{512}=\frac{1}{n}3.71\;µm/\mathrm{pixel}=2.65\;µm/\mathrm{pixel}=0.00265\;\mathrm{mm}/\mathrm{pixel}$$

#### 2.6.4. Textural Properties

Differences in the epidermis and dermis granularity of OCT images were observed. Hence, textural feature analysis could be of great value for lesion differentiation. By performing textural analysis on the ROI flattened sub-image, it was possible to obtain a set of features that could be quantified and compared. Thus, first, the Gray Level Co-occurrence Matrix (GLCM) [33] was calculated with a distance “d” for the angles 0°, 90°, 180°, and 270°. Afterwards, contrast, dissimilarity, homogeneity, energy, and correlation features were calculated. The variable “d” indicated the distance between the pair of pixels in the image and the angles with the directions to consider on the calculation of the GLMC. Contrast identified the differences in brightness between elements and the background and it was also known as inertia. Dissimilarity detected local variations, measuring distances between the objects. Homogeneity was associated with the smoothness of the image texture and measured the density (closeness) of element distribution. Energy was related with the uniformity of the image, where a high value indicated high uniformity of the image. Finally, correlation calculated linear dependencies in the image.

ROI sub-images were divided in smaller boxes and iteratively analysed to obtain the selected textural features. Boxes of sizes 10 × 10 px (~70 × 26 µm), 20 × 20 px (~140 × 52 µm), 25 × 25 px (~175 × 66 µm), and 50 × 50 px (~350 × 132 µm), for two different distances, (d = 2 and d = 5) were calculated and the results were compared.

## 3. Results

Optical and textural properties were estimated for all the case studies. The histopathological HE slices for the study of further features and comparison with the OCT images are included in Appendix A. This appendix also includes a table that collected main features observed in the HE images defined with the support of a pathologist. 

### 3.1. Optical Properties

**Figure 7:**
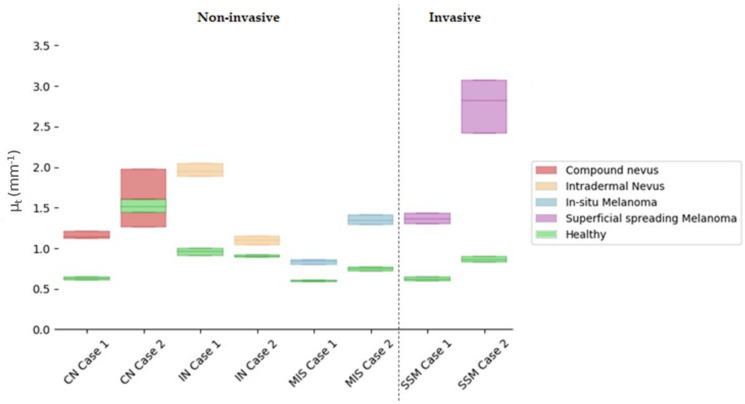
Comparison of quartile µ_t_ values for selected cases’ lesion tissues versus healthy adjacent tissues in the lesion centre. Y axis illustrates the µ_t_ value and its corresponding IQR interquartile ratio, where the middle line represents the median. X axis orders the different case studies grouped by colour as per diagnosis.

Attenuation coefficients (µ_t_) were calculated for the ROI automatically extracted from the lesion centre and compared with the healthy tissue adjacent to the lesion. The ROI analysed had 100 px width and 150 px depth (~0.7 × 0.4 mm). The lesion centre was considered by default as the more representative part of the lesion, as it happened to be in some of the diagnosis under study, for example, in the case of compound nevus, where the borders presented characteristics associated with a different diagnosis. Figure 7 illustrates the obtained results, where values were limited to the interquartile ratio (IQR) to facilitate the interpretation. Different colours were assigned to the case study diagnosis, wherein green colour was assigned to healthy adjacent tissue and indicated in the figure legend. Values are collected in detail in Table 6 for the lesions and the healthy adjacent tissue.

All things considered and given the reported results of the calculated µ_t_ obtained with the current case studies’ data, the answers to the questions raised at the beginning of this work would be the following:

**Answer to Question 1:** SSM Case 1 was in the value range of other lesion types (overlapping), making the differentiation not feasible. However, SSM Case 2 stood out for being superior. A possible explanation for this could be that SSM Case 2 had a severe inflammatory response when compared to SSM Case 1, where inflammation was mild. 

**Answer to Question 2:** Focusing only on the total µ_t_ value, it was possible to observe an overlap of MIS Case 2 and SSM Case 1. The reason behind this could be that SSM Case 1 was an early-stage melanoma (AJCC: IA), and hence, it was thin with very mild inflammatory response as the case with MIS Case 2. Both lesions also presented with the same melanocyte pattern in the epidermis and pigmentation level both in the epidermis and dermis. However, when quantifying the difference between the MIS and SSM lesions and the healthy reference, it was possible to observe a clear separation between both types of lesions.

**Answer to Question 3:** There was an overlap between CN Case 2 and SSM Case 1. CN Case 2 had a moderate dermal component, increasing the lesion scattering and hence the total attenuation factor. 

**Answer to Question 4:** Focusing only on the total µ_t_ value, it was possible to observe an overlap of values. Both IN cases had a moderate to severe dermal component. On the other hand, IN Case 1 was an acral lesion which poses a difference in the total attenuation of the lesion. However, when analysing the difference with respect to their healthy reference, separation between subtypes was observed.

In general terms, answering previous questions seemed difficult when only relying on the total attenuation factor µ_t_. However, some promising findings were encountered, and the µ_t_ values were in the expected ranges as previously reported [14], serving as a confirmation of the possibilities of this optical property. As observed in Appendix A, HE images and Table A1, the case studies contained different causative factors (such as the level of inflammatory response, pigmentation level, or dermal component) that may influence both the absorption and scattering of the signal. In this sense, a more detailed analysis calculating both factors separately and a larger number of cases would be necessary to confirm the findings glimpsed in this work. 

Differences between the lesion and healthy adjacent tissue were noticeable wherein the lesion µ_t_ was always higher than the adjacent healthy µ_t_. The exception in this case was in CN Case 2 that showed an overlap between the lesion and healthy µ_t_ values. Observing the corresponding dermatoscopic image in Table 2, it was not clear whether what was considered as healthy adjacent tissue in the OCT image was really “totally” healthy tissue due to the lighter pigmentation in the borders. 

### 3.2. Textural Properties

After careful textural analysis optimization, the most noteworthy results and differences between lesions and healthy tissue were found, using a distance d = 2 and 25 × 25 pixels (~175 × 66 µm) box size, after the calculation of the properties using the GLMC. The view in depth was limited to 75 px, examining a total ROI area of 100 × 75 px (~0.7 × 0.2 mm) in the lesion. The three sections in depth were partitioned as follows: from 0 to 25 px, 25 px to 50 px, and 50 px to 75 px. A fourth section from 50 px to 100 px was also studied but was finally discarded since it did not provide additional information of interest with impact on the diagnosis.

**Figure 8:**
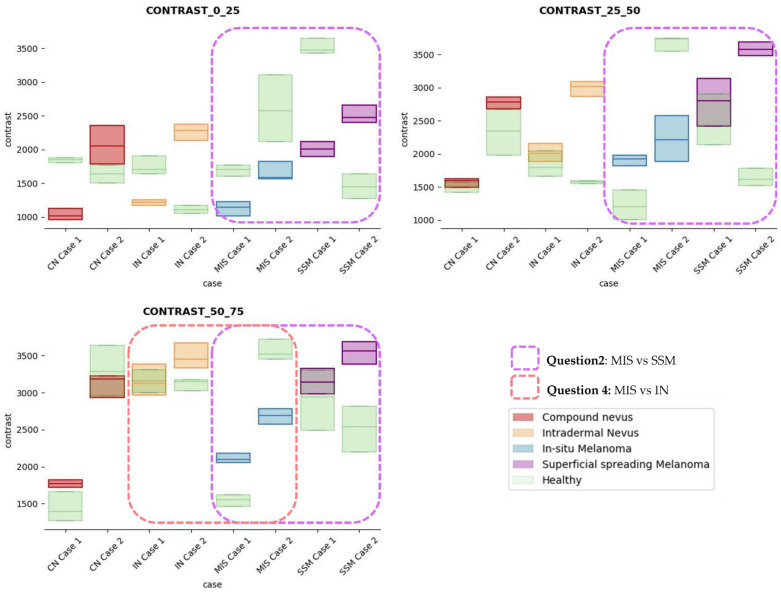
Contrast textural feature comparisons for case studies’ lesions with respect to adjacent healthy tissue (left: 0–25 pixels in depth; centre: 25–50 pixels in depth; right: 50–75 pixels in depth).

**Figure 9:**
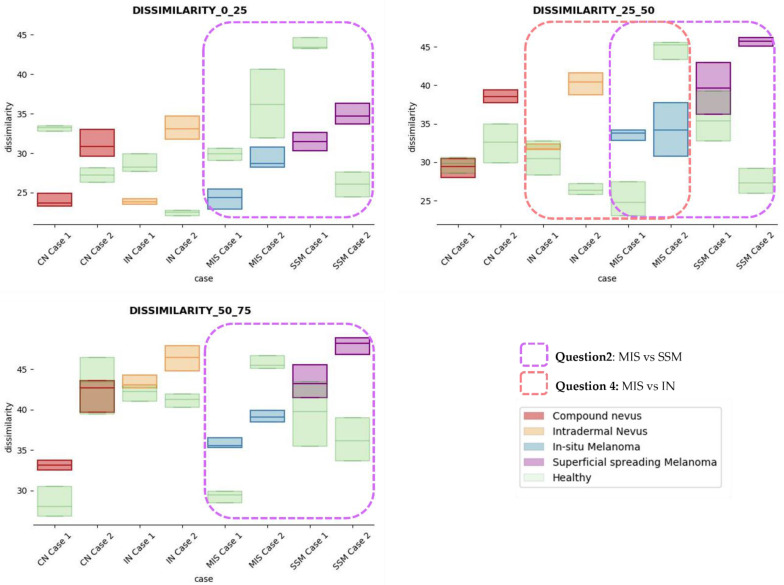
Dissimilarity textural feature comparisons for case studies’ lesions with respect to adjacent healthy tissue (left: 0–25 depth; centre: 25–50 depth; right: 50–75 depth).

**Figure 10:**
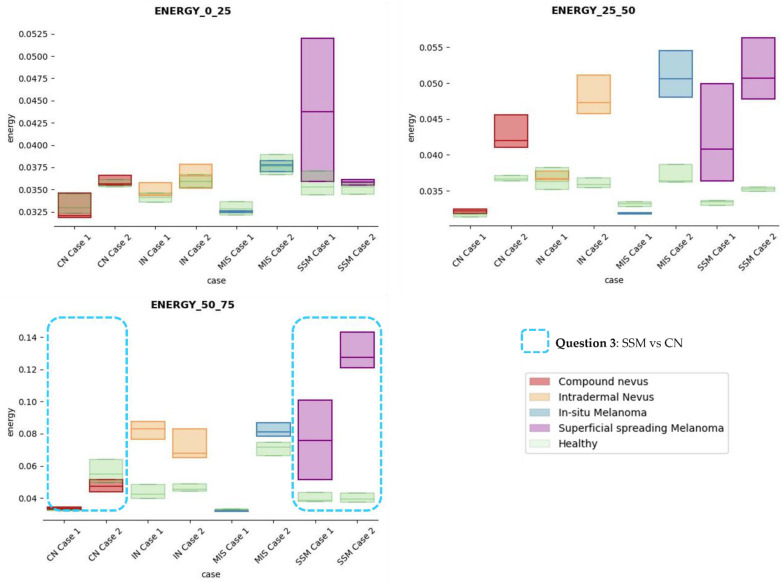
Energy textural feature comparisons for case studies’ lesions with respect to adjacent healthy tissue (left: 0–25 pixels in depth; centre: 25–50 pixels in depth; right: 50–75 pixels in depth).

**Figure 11:**
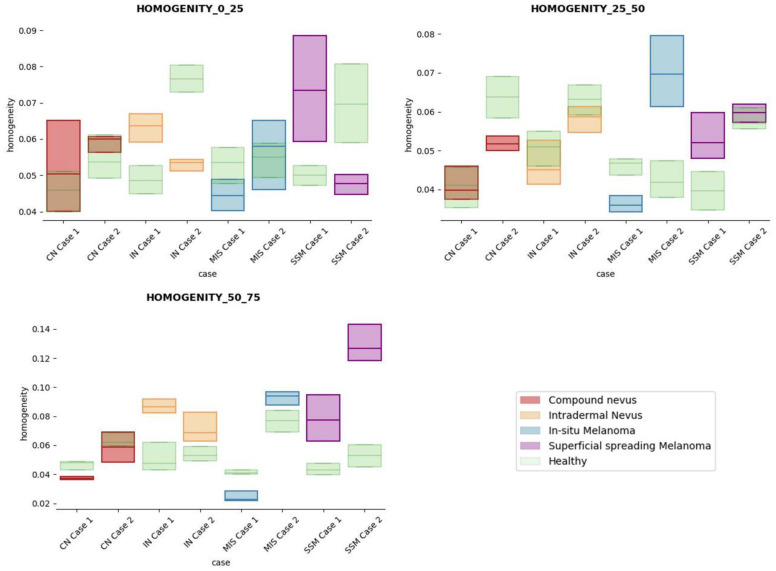
Homogeneity textural feature comparisons for case studies’ lesions with respect to adjacent healthy tissue (left: 0–25 pixels in depth; centre: 25–50 pixels in depth; right: 50–75 pixels in depth).

**Figure 12:**
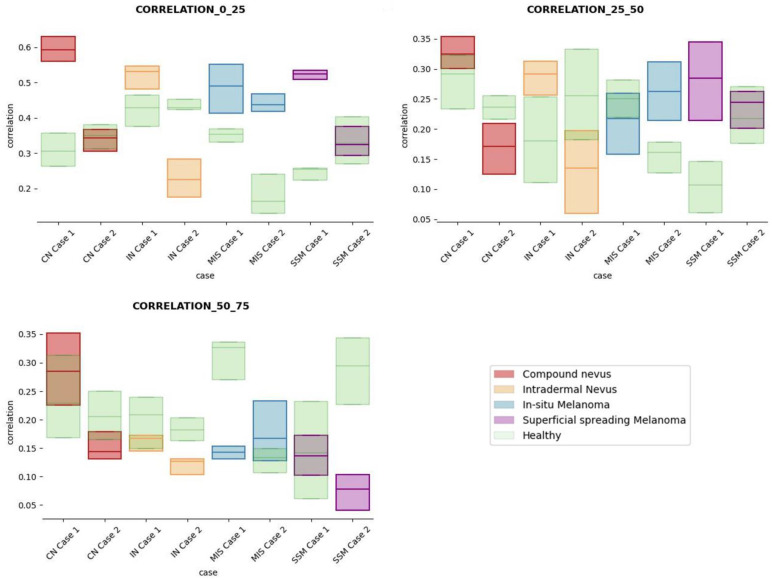
Correlation textural feature comparisons for case studies’ lesions with respect to adjacent healthy tissue (left: 0–25 pixels in depth; centre: 25–50 pixels in depth; right: 50–75 pixels in depth).

The results obtained for the three sections were studied in detail studied for the four questions formulated in this work. Not only were the textures inside the lesion ROI studied, but also those related to the adjacent healthy tissue as performed with the optical properties. The results for the contrast feature are presented in Figure 8, dissimilarity in Figure 9, energy in Figure 10, homogeneity in Figure 11, and correlation in Figure 12. Features’ figures were sorted in the order of relevance. 

After textural analysis, the answers to the questions raised in this work are presented below: 

**Answer to Question 1:** This generic classification did not seem to be feasible at this stage of development. However, it was possible to observe large differences in SSM lesions with respect to adjacent healthy tissue in contrast, dissimilarity, and homogeneity features in the most superficial layers of the skin (first section, left column of figures).

**Answer to Question 2:** The contrast and dissimilarity features (see Figure 8 and Figure 9) showed interesting differences between MIS and SSM for all the three sections analysed. These features correctly identified the alterations that occurred in the epidermis and dermis layers in the presence of tumour and, more specifically, also the differences between nevus and melanoma intrinsic alterations (as summarized in Table A1).

**Answer to Question 3:** The energy feature has potential in the separation of the SSM and CN diseases, as differences were observed in the first and third sections, especially in the deeper area (right column in Figure 11). This makes sense because both lesions present alterations in the dermal layer. CN lesions are typically symmetric and should be more uniform than SSM, which are more heterogenous and chaotic and with more alterations of elements that affect tissue uniformity.

**Answer to Question 4:** The contrast and dissimilarity features (see Figure 8 and Figure 9) showed interesting differences between MIS and IN in the deeper region of analysis (third section, right columns) as changes in the dermis for the IN cases were clearly detected.

In summary, it is worth mentioning that the contrast and dissimilarity features have a great potential for the differentiation of the different diseases, as they have demonstrated that they are able to identify the alterations that happens in the presence of tumour and even the variations between nevus and melanoma. Differences with respect to healthy tissue were also observed in these properties, especially for MIS and SSM lesions, where homogeneity feature also plays a role. Energy has also demonstrated potential for the differentiation of SSM versus CN and deserves further study.

## 4. Discussion and Conclusions

OCT technology has been studied in the latest years as a supporting imaging technique in the dermatological clinical practice. Although its full potential has been demonstrated in diagnosing non-melanoma skin cancer [34], its potential in the diagnosis of melanoma lesions remains unsolved [35]. Image interpretation is not easy for untrained clinicians (with high subjectivity); thus, some artificial intelligence analysis strategies are being explored. Unfortunately, most pursued strategies pretend to create generic solutions that are able to distinguish between a vast number of lesions; for example, one of the main concerns being that melanoma is misled as basal cell carcinoma in some cases. All these problems prevent the adoption of OCT technology in clinical practice. However, perhaps the problem relies on such a generic approach, and the solution should be focusing the efforts on building more specific tools for more limited problems. Artificial intelligence solutions that are able to generalize are long being pursued. However, in the medical field, they are not realistic given the high heterogeneity of the diseases, especially at the histopathological level, as it is in the case with melanoma [36], which is known as the “great mimic”.

The aim of this work was to maximize the usefulness of the information present in OCT images for the diagnosis of melanoma, targeting the differentiation between non-invasive and invasive lesions, as this is a critical need demanded by the dermatologists involved in this study. With this motivation and the final future goal of providing a CADx tool for OCT automatic image analysis and diagnosis support, this work performed a study of various selected cases studies. Optical and textural properties were extracted and studied from OCT images in comparison with histopathological HE images and features extracted by an expert pathologist. This required selection of OCT and HE images for each lesion that could be compared (implying that the biopsy paraffin cut is parallel to the OCT scanning path and hence “co-registered” and comparable), limiting the number of usable cases available for analysis but which made it possible to find the histopathological reasons (Table A1) behind the alterations. Previous works have also considered the study of image features [15], histopathological features [16], or optical features [18,19] separately and not in an integrated manner.

A series of four clinically oriented questions were raised at the beginning of this work to serve as a guide in the analysis of the extracted features and, more importantly, to identify the future directions of development towards the final goal. In this sense, it can be concluded that there is still an important way to go to be able to create a supportive diagnostic tool that is able to automatically distinguish non-invasive versus invasive lesions. However, interesting findings and promising results were identified during the analysis of the extracted properties on the differentiation of melanoma in-situ (non-invasive) versus superficial spreading melanoma (invasive) and this deserves further study. The viabilities of differentiating superficial spreading melanoma versus compound nevus (both in the epidermis and dermis) and melanoma in-situ (epidermis) versus intradermal nevus (dermis) have been also studied and have revealed possibilities in their differentiation. These problems are very interesting from the automatic image processing point of view, but they also have a clinical impact and contribute to the final goal. Additional analysis with more samples will be necessary to confirm the suitability of the proposed approach.

As future steps in the short term, optical features should be studied in more detail. The histopathological features analysed in the appendix identify elements such as the pigmentation level (in the epidermis and dermis), dermal component, inflammatory response, etc., all of which influence the absorption and scattering of the lesion. Hence, ideally, the signal should be disaggregated into absorption (µ_a_) and scattering (µ_s_) to be studied separately. Additionally, the textural feature studies have revealed interesting findings as complementary information, but results need to be validated with more examples from with other studies of the SoA. For all the extracted features, the observed differences of the lesion tissue with respect to healthy adjacent tissue also deserve some attention. Further, the anatomical differences between the epidermis and dermis layers due to body location [37], relaxation vs. overextension of muscles and limbs [38], or aged and damaged skin [39] could have an effect and deserve more study, as, for example, seen in the IN Case 1 sample. 

Later on, the goal is to integrate the results in a deep learning-based solution where more implicit lesion features can be automatically extracted. Image processing and machine learning tools are becoming increasingly sophisticated, demonstrating their validity during diagnosis with success rates comparable to expert clinicians [40]. Previous test performed by the authors revealed that well-known deep learning models working directly on OCT B-scans were not good enough for aided diagnosis with the collected samples. However, in the latest years, hybrid models are gaining relevance in the medical field, and a strategy based on combining various features (also clinical) together with dermatoscopic and OCT images in a deep learning model must be pursued. In this sense, dedicated studies to demonstrate the validity of the approach in daily medical practice will be necessary.

## Figures and Tables

**Table 1:** Skin layer alterations for the lesion types and sub-types considered in the study.

	Epidermis	Dermis
**Non-invasive**		
Compound nevus (CN)	X	X
Intradermal nevus (IN)		X
Melanoma in-situ (MIS)	X	
**Invasive**		
Superficial spreading melanoma (SSM)	X	X

**Table 2:** Overview of dermatoscopy and OCT images of compound Nevus (CN) case studies (non-invasive).

Compound nevus (CN) Case 1	Localization: right dorsal
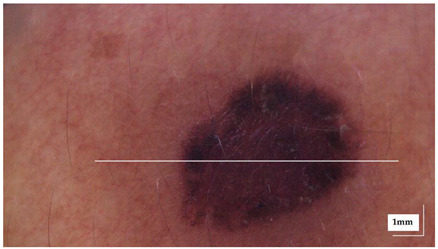	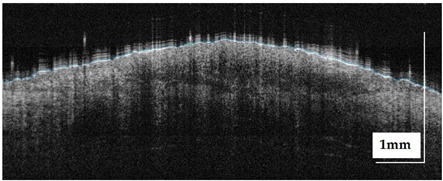
Compound nevus (CN) Case 2	Localization: right posterior thigh
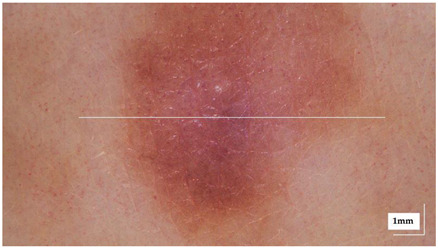	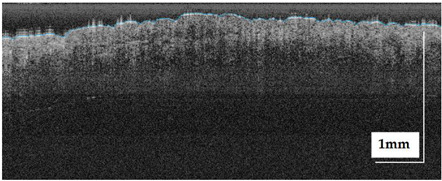

**Table 3:** Overview of dermatoscopy and OCT images of intradermal nevus (IN) case studies (non-invasive).

Intradermal nevus (IN) Case 1	Localization: right forefoot (sole)
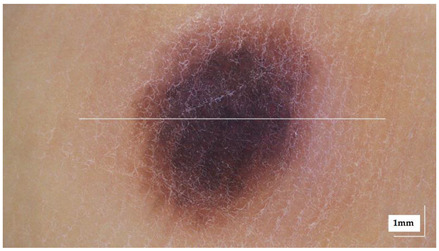	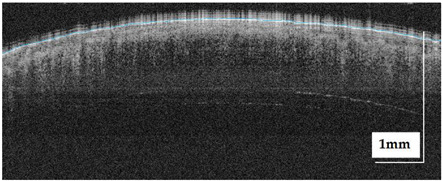
Intradermal nevus (IN) Case 2	Localization: left dorsal
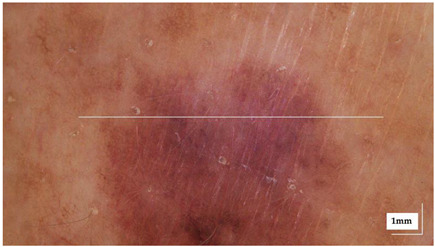	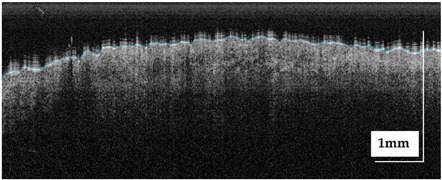

**Table 4:** Overview of dermatoscopy and OCT images of melanoma in-situ (MIS) case studies (non-invasive).

Melanoma in-situ (MIS) Case 1	Localization: right abdomen
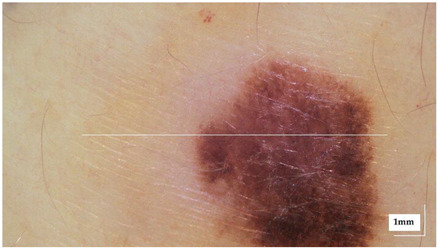	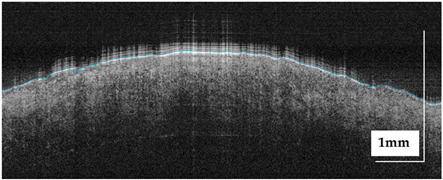
Melanoma in-situ (MIS) Case 2	Localization: left chest
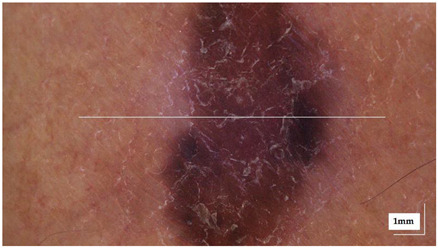	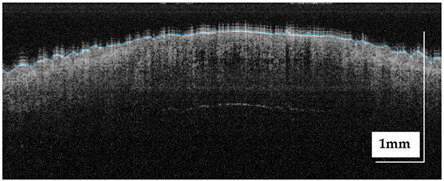

**Table 5:** Overview of dermatoscopy and OCT images of superficial spreading melanoma (SSM) case studies (invasive).

Superficial spreading melanoma (SSM) Case 1	Localization: right anterior arm
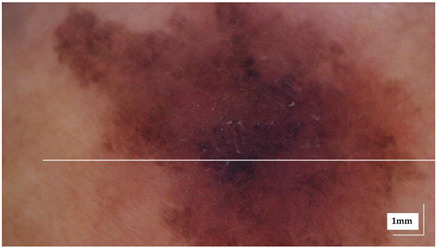	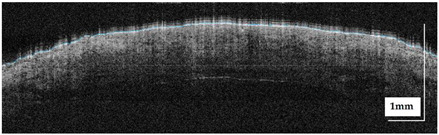
Superficial spreading melanoma (SSM) Case 2	Localization: right forefoot (right ankle)
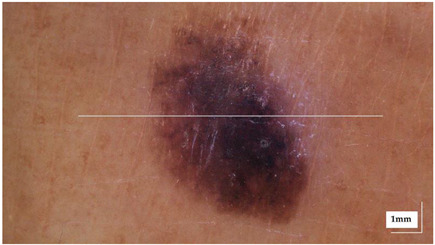	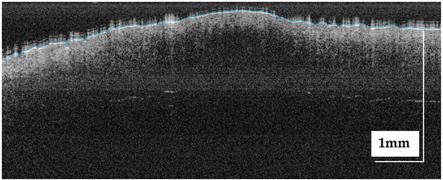

**Table 6:** Attenuation (µ_t_) coefficients for selected cases’ lesion tissue in the centre of the lesion (mm^−1^) with respect to adjacent healthy tissue.

	LESION	HEALTHY (Adjacent)	
Non-Invasive	Median—IQR ^1^ (mm ^−1^)	Median—IQR (mm ^−1^)	Median Difference
CN Case 1	1.14–0.10	0.62–0.04	0.52
CN Case 2	1.60–0.71	1.52–0.16	0.08
IN Case 1	1.94–0.15	0.96–0.09	0.98 ^2^
IN Case 2	1.10–0.11	0.90–0.03	0.20
MIS Case 1	0.84–0.06	0.60–0.03	0.24
MIS Case 2	1.34–0.13	0.75–0.05	0.59
**Invasive**			
SSM Case 1	1.37–0.13	0.62–0.05	0.75
SSM Case 2	2.82–0.65	0.86–0.08	1.96

^1^ IQR = Inter-Quartile Ratio; ^2^ Note that this is an acral lesion: thicker stratum corneum and epidermis (see Figure A3).

**Figure A1:**
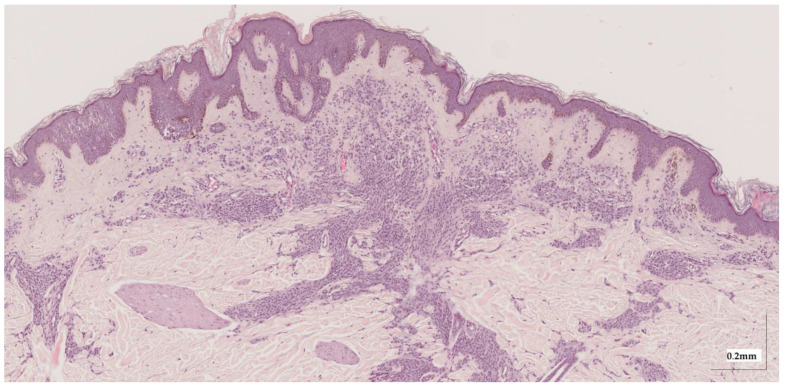
Complete HE slide of compound nevus Case 1 (20× magnification).

**Figure A2:**
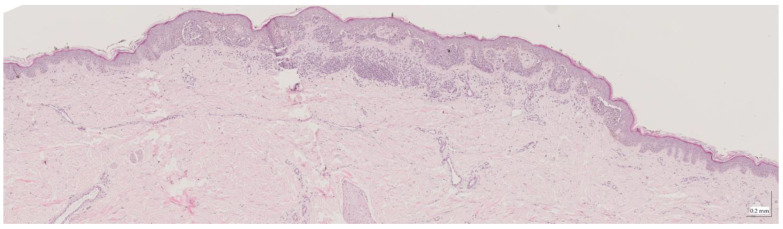
Complete HE slide of compound nevus Case 2 (20× magnification).

**Figure A3:**
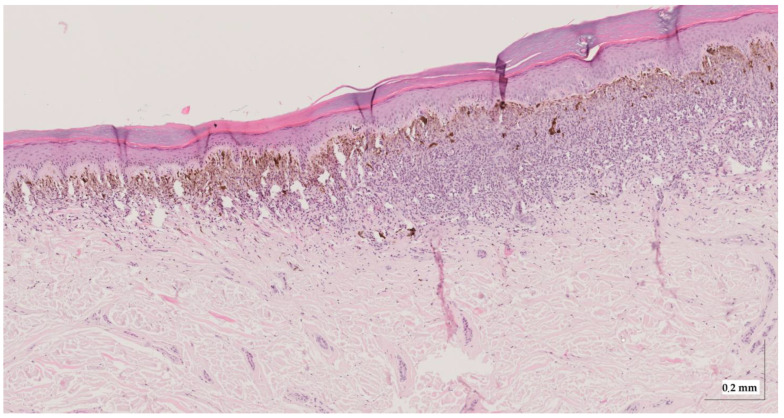
Complete HE slide of intradermal nevus Case 1 (20× magnification).

**Figure A4:**
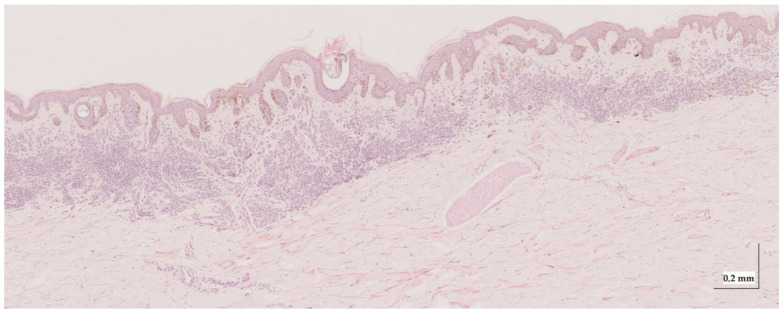
Complete HE slide of intradermal nevus Case 2 (10× magnification).

**Figure A5:**
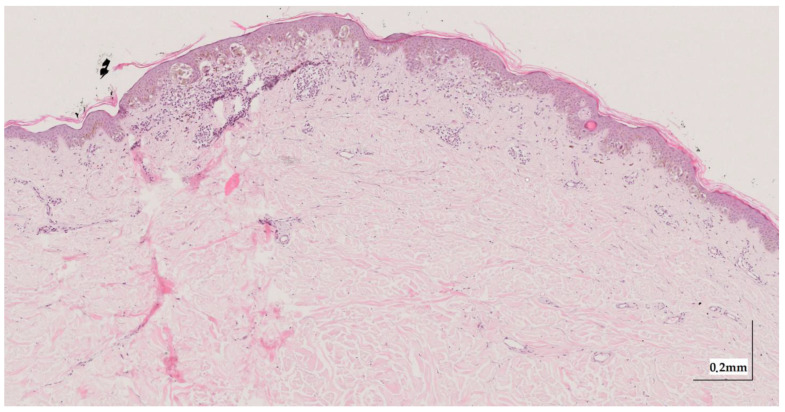
Complete HE slide of melanoma in-situ Case 1 (10× magnification).

**Figure A6:**
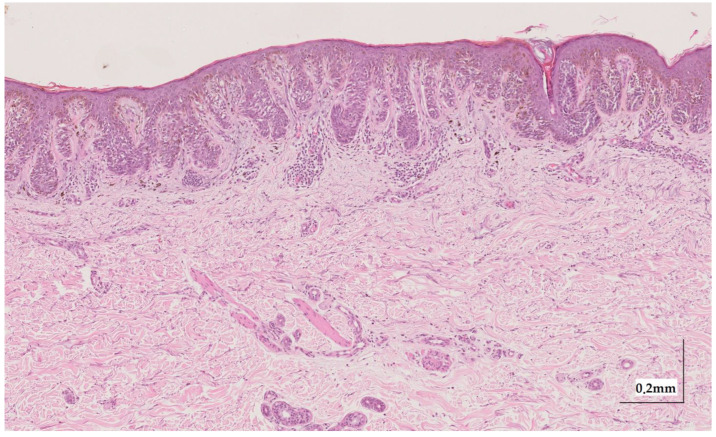
Complete HE slide of melanoma in-situ Case 2 (10× magnification).

**Figure A7:**
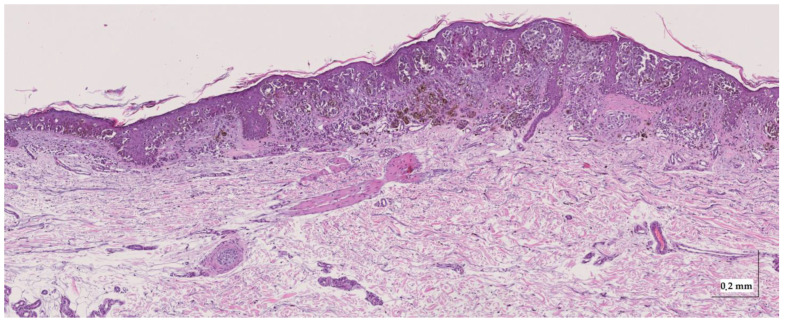
Complete HE slide of superficial spreading melanoma Case 1 (10× magnification).

**Figure A8:**
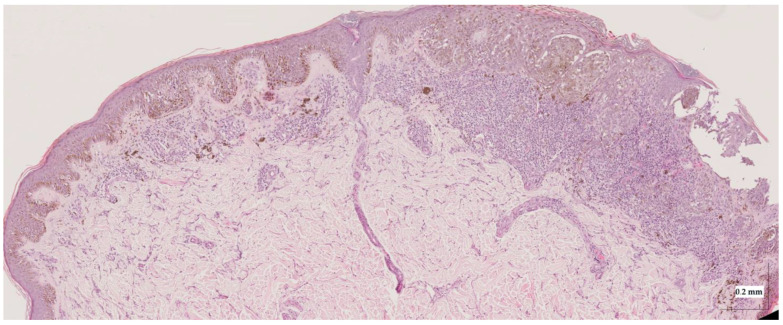
Complete HE slide of superficial spreading melanoma Case 2 (20× magnification).

## Data Availability

Data available on request due to restrictions such as privacy or ethics. The data presented in this study are available on request from the corresponding author. The data are not publicly available since approval from the ethical committee is necessary for each request as data belong to real patients who have signed informed consent only for the current study.

## Appendix A. Selected Cases Studies’ HE Histopathological Images (Limited to OCT FOV)

This appendix includes the HE digitalized slides of the cases studies under study for comparison study and facilitate the localization of histopathological properties. HE images have been edited so that attention can be directed to the region of the image where the lesion is located on the OCT images (as described in Section 2.6.1. “Automatic lesion delimitation”) and hence avoid confusion when deeper elements are present in the HE slides.

This appendix also includes Table A1 where the main characteristics observed in the HE images have been collected with the support of an expert pathologist.

**Table A1:** Summary of characteristics observed in the selected cases’ HE images.

	Pigmentation EPIDERMIS	Pigmentation DERMIS	Inflammatory Answer	Melanocytes’ Pattern EPIDERMIS	Atypia	Growing Pattern	Dermal Component
CN1	Moderate	None–Mild	N.A.	Isolated + 1 net	N.A.	N.A.	Severe
CN2	Mild	None–Mild	N.A.	Nets + Lentiginous	N.A.	N.A.	Moderate
IN1	None	Severe	N.A.	N.A.	N.A.	N.A.	Moderate–Severe (centre-right) Mild (left)
IN2	Moderate	Mild	N.A.	N.A.	N.A.	N.A.	Moderate–Severe
MIS1	Moderate	None	Mild (right) Moderate (left)	Nets	Moderate– Severe	N.A.	N.A.
MIS2	Moderate	Moderate (and perivascular)	Mild	Nets + Lentiginous	Mild–Moderate	N.A.	N.A.
SSM1	Moderate	Moderate–High (centre)	Mild	Nets + Lentiginous	Moderate–Severe	Horizontal	N.A.
SSM2	Severe (left) + Moderate (right)	Moderate	Severe (right) + Moderate (left)	Lentiginous (left) + Nets (centre, right)	Mild–Moderate (left), + Severe (right)	Horizontal	N.A.
